# 4.6: Anisotropy in solid breast lesions at shear wave elastography: relationship to the radial plane and implications for benign/malignant differentiation

**DOI:** 10.1186/bcr3502

**Published:** 2013-11-08

**Authors:** K Skerl, S Vinnicombe, P Whelehan, K Thompson, D McLean, A Evans

**Affiliations:** 1 University of Dundee, UK

## Introduction

Anisotropy is the directional dependence of the measurement of a property. As breast tissue structure and some breast diseases (DCIS) are anisotropic in structure, we aimed to establish the frequency, degree and diagnostic value of shear wave elastography anisotropy in solid breast lesions.

## Methods

Ninety-eight solid breast lesions (31 benign and 67 malignant) were examined in the radial and anti-radial planes, with two mean stiffness measurements (in kPa) being taken in each plane and averaged. The difference between the radial and anti-radial measurements was squared to make all readings positive, and compared with the histological diagnosis. Paired Student *t *tests and chi-square tests were performed to establish statistical significance of the relationships.

## Results

Anisotropy was found in both benign and malignant lesions. However, the stiffness values were not related to the examination plane in either group of lesions (*P *= 0.2). Anisotropy was greater in malignant lesions than benign lesions (*P *< 0.0001). Using a malignancy threshold value for the square of the difference in radial and anti-radial stiffness of 200, the sensitivity, specificity and diagnostic accuracy of the presence of this level of anisotropy were 72%, 87% and 77% respectively.

## Conclusion

Stiffness of solid breast lesions on shear wave elastography is not directly related to the examination plane. Malignant lesions show more anisotropy than benign lesions. Therefore, adding anisotropy to other shear wave parameters has the potential to improve the ability of this modality to differentiate benign from malignant solid breast lesions.

